# Spontaneous CD4+ T Cell Activation and Differentiation in Lupus-Prone B6.Nba2 Mice Is IFNAR-Independent

**DOI:** 10.3390/ijms23020874

**Published:** 2022-01-14

**Authors:** Emma J. Keller, Nina Dvorina, Trine N. Jørgensen

**Affiliations:** 1 Department of Molecular Medicine, Cleveland Clinic Lerner at Case Western Reserve University, Cleveland, OH 44195, USA; ejk114@case.edu; 2 Department of Inflammation and Immunity, Lerner Research Institute, Cleveland Clinic, Cleveland, OH 44195, USA; dvorinn@ccf.org

**Keywords:** SLE, interferon-alpha receptor, T cell, SLE, CD8+

## Abstract

Systemic lupus erythematosus (SLE) is an autoimmune disorder characterized by dysregulated T and B lymphocytes. Type I interferons (IFN-I) have been shown to play important pathogenic roles in both SLE patients and mouse models of lupus. Recent studies have shown that B cell intrinsic responses to IFN-I are enough to drive B cell differentiation into autoantibody-secreting memory B cells and plasma cells, although lower levels of residual auto-reactive cells remain present. We speculated that IFN-I stimulation of T cells would similarly drive specific T-cell associated lupus phenotypes including the upregulation of T follicular helper cells and Th17, thereby affecting autoantibody production and the development of glomerulonephritis. Using the B6.Nba2 mouse model of lupus, we evaluated disease parameters in T cell specific IFN-I receptor (IFNAR)-deficient mice (cKO). Surprisingly, all measured CD4+ T cell abnormalities and associated intra-splenic cytokine levels (IFNγ, IL-6, IL-10, IL-17, IL-21) were unchanged and thus independent of IFN-I. In contrast B6.Nba2 cKO mice displayed reduced levels of effector CD8+ T cells and increased levels of Foxp3+ CD8+ regulatory T cells, suggesting that IFN-I induced signaling specifically affecting CD8+ T cells. These data suggest a role for both pathogenic and immunosuppressive CD8+ T cells in *Nba2*-driven autoimmunity, providing a model to further evaluate the role of these cell subsets during lupus-like disease development in vivo.

## 1. Introduction

Systemic lupus erythematosus (SLE) is an autoimmune disorder in which immune cells and self-reactive antibodies drive multiple organ inflammation and damage. Albeit being a B cell-driven disease, dysregulation within the T cell compartment has been reported in both SLE patients and mouse models of lupus. As such, SLE patients display increased levels of CD4+ Th17 cells, with concomitantly downregulated CD4+ Th1 and FOXP3+ CD4+ Treg cells [1,2]. Abnormal cell subsets, such as CD4-CD8- double negative (DN) T cells and CD8+CD28- T cells have been identified in SLE patients and suggested be the main producers of IL-17 and IL-10, respectively [3,4,5]. Furthermore, levels of IL-17, IL-10 and IFNγ have been found to be increased in SLE patients [1,6], the latter predominantly in patients with active nephritis [7]. Finally, SLE patients present with increased populations of circulating IL-21-producing T follicular helper (Tfh) cells, essential for germinal center (GC)-driven B cell differentiation and autoantibody production [8,9,10].

In mouse models of lupus, activated T cells and increased frequencies of effector/memory CD4+ T cells have been reported repeatedly [11,12,13,14,15]. Moreover, MHC class II-deficient lupus-prone mice, which have significantly decreased populations of CD4+ T cells, were protected from disease development [16], strongly supporting a role for CD4+ T cells in disease pathogenesis. Within the CD4+ T helper cell population, both Th1 and Th17 cells and their cytokine products, IFNγ and IL-17, have been found to be elevated relative to Treg cells in lupus-prone mouse models [17,18,19]. Whether Th1 and Th17 cells are specifically associated with organ inflammation remains unclear, although circumstantial evidence from IL-2-treated MRL/lpr mice suggests that a shift in the Th17:Treg balance favoring less Th17 cells is enough to reduce renal damage in this model [18]. IFNγ, the main cytokine produced by Th1 cells, has been found to be upregulated in most spontaneous lupus mouse models including (NZB×NZW)F1, B6.Sle1b and MRL/lpr [14,20]. Recent data suggest that IFNγ contributes specifically to nephritis, as IFNγR1-deficient Sle1b.Yaa mice present with reduced glomerular nephritis scores compared to both B6.Sle1 and Sle1.Yaa mice [21]. Finally, Tfh cells are essential for functional GC formation and elevated levels of Tfh cells have been found in most lupus-prone mouse models, including B6.Nba2 mice [12]. Not surprisingly, Tfh-deficiency protects lupus-prone Sanroque mice from disease development [22]. Furthermore, Tfh cells interact with GC B cells via CD40/CD40L and ICOS/ICOSL interactions and blocking of either costimulatory pathway reduces lupus-like disease in (NZB×NZW)F1 and SNF1 mice [23,24].

Type I interferons (IFN-I) are involved in disease pathogenesis in most mouse models of lupus [25,26,27,28,29,30], and an IFN-I-induced gene expression signature has been described in SLE patients numerous times [31,32]. IFN-I has been shown to directly target T cells in several settings. In vitro, IFN-I were shown to prolong survival of CD4+ T cells [33], and in a non-lupus model, IFN-I enhanced the clonal expansion of antigen-specific CD4+ T cells by increasing survival signals and leading to increased numbers of IFNγ producing cells [34]. Similarly, the survival of IFNAR-deficient CD8+ T cells after viral antigen exposure was significantly diminished compared to WT CD8+ T cells [35]. There is also evidence that IFN-I stimulation facilitates cross-priming of CD8+ T cells [36]. Finally, studies have shown that IFN-I induces increased expression of *Bcl6*, the master transcription factor for Tfh cells [37], suggesting a key role for IFN-I in facilitating GC reactions.

Although rarely described as a main contributor to SLE pathogenesis, CD8+ T cells are known to be dysregulated in SLE patients. In particular, a specific population of exhausted CD8+ T cells (PD1^high^IL-7R^low^) were identified in subsets of SLE patients and associated with a reduction in flares [38]. In animal models of lupus, a role for CD8+ T cells was first identified in (NZB×NZW)F1 mice, which upon treatment with depleting anti-CD8 antibodies developed accelerated disease, thus suggesting that at least a subset of CD8+ T cells exert immunosuppressive functions [39,40]. Newer studies revealed that CD8+ Foxp3+ Tc regulatory cells suppressed effector T cell proliferation and autoantibody production by lupus B cells in vitro in a Foxp3- and PD1-dependent manner [41]. PD-1 has also been identified as a marker for exhausted CD8+ T cells present at elevated levels in nephritic kidneys of 25 weeks old MRL/lpr mice, when compared to pre-nephritic animals [42]. Interestingly, expression of the interferon-inducible protein Ifi202b, encoded within the *Nba2* congenic region [43], was found to drive the suppressive activity of CD8+ Tc regulatory cells via upregulation of Foxp3, TGFβ and IL-2 in (NZB×NZW)F1 mice [44].

In this study, we investigated the role of IFN-I on T cells in B6.Nba2 lupus-prone mice, hypothesizing that IFN-I promote lupus-like disease in this model by enhancing pathogenic T cell populations, such as Th1, Th17 and Tfh cells, providing cytokines and/or direct help to bolster an autoimmune response. In order to determine if direct IFN-I stimulation of T cells drive pathogenicity, we generated T cell specific IFN-I receptor (IFNAR) conditional knock out mice (B6.Nba2.TΔIFNAR) (from here on named B6.Nba2 cKO). Interestingly, we found that the extensive dysregulation of the CD4+ T cell compartment in B6.Nba2 mice was independent of IFN-I stimulation. In contrast, an *Nba2*-driven accumulation of CD44^high^CD62L^low^ effector CD8+ T cells is partly reversed by T-cell specific IFNAR-deficiency, while a population of Foxp3+ CD8+ Tc regulatory cells was found to be upregulated in B6.Nba2 cKO mice. Thus, T-cell specific IFNAR deficiency affects select CD8+ T cell subsets, with minimal effect on CD4+ T cells in B6.Nba2 mice.

## 2. Results

### 2.1. T-Cell Specific IFNAR Deficiency Does Not Affect Splenic CD4 and CD8 T Cell Dysregulation in B6.Nba2 Mice

**Figure 1:**
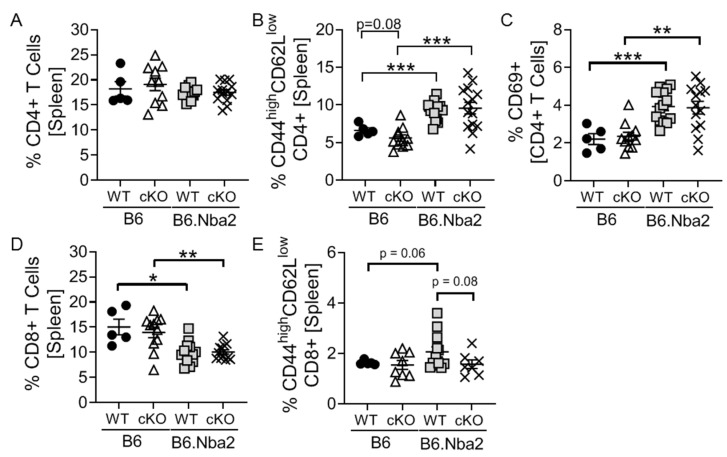
Dysregulated CD4+ and CD8+ in B6.Nba2 mice are independent of T-cell intrinsic IFNAR expression. IFNAR-sufficient and -deficient female B6 and B6.Nba2 mice were analyzed for frequencies of CD4+ and CD8+ cells at 4 months of age by flow cytometry. (**A**) Total splenic CD4+ T cells, (**B**) CD44highCD62Llow effector/memory CD4+ T cells, (**C**) CD69+ recently activated CD4+ T cells, (**D**) Total CD8+ T cells, (**E**) CD44highCD62Llow effector/memory CD8+ T cells. Each symbol represent one mouse. *n* = 5 (B6), *n* = 11 (B6 cKO), *n* = 14 (B6.Nba2), *n* = 9 (B6.Nba2 cKO). * *p* < 0.05; ** *p* < 0.01; *** *p* < 0.001, Student’s unpaired *t*-test with Welch’s correction.

Since it is well established that lupus-like disease in the B6.Nba2 mouse model depends on functional type I interferon (IFNAR) expression [25], we evaluated the specific role IFN-I stimulation of T cells play in disease using T-cell specific IFNAR conditional knock out animals. Lack of functional IFNAR expression on T cells (CD4+ and CD8+) was verified upon stimulation with recombinant IFNα in vitro (see Figure S1). At 4 months of age, control C57Bl/6 (B6) and B6.Nba2 mice, either expressing IFNAR (WT) or not (cKO), were sacrificed and frequencies of T cell subsets were evaluated. There were no difference in levels of CD4+ T cells between B6, B6 cKO, B6.Nba2 and B6.Nba2 cKO mice (Figure 1A). Confirming our previous observations, B6.Nba2.WT animals displayed significantly elevated levels of CD4+ effector/memory T cells (CD44^high^CD62L^low^) as well as recently activated T cells (CD69+) (Figure 1B,C). This upregulation was, however, not driven by IFNAR expression on T cells, as neither cell population was different between B6.Nba2 and B6.Nba2 cKO mice (Figure 1B,C). We found significantly reduced levels of total CD8+ T cells in B6.Nba2 mice as compared with B6 mice, but again no difference between IFNAR-sufficient and IFNAR-deficient CD8+ T cells in B6.Nba2 mice (Figure 1D). Further analysis of CD8+ T cell subsets, showed a trend towards increased levels of CD44^high^CD62L^low^ CD8+ T cells in B6.Nba2 versus B6 mice, which was partly reversed in B6.Nba2 cKO mice (Figure 1E). Thus, while the *Nba2* locus drives spontaneous activation of CD4+ T cells and a reduction of total CD8+ T cells, only the accumulation of effector CD8+ T cells appears to be driven by IFNAR expression on T cells.

### 2.2. B6.Nba2 Mice Accumulate Th1 and Th17 Cells in an IFNAR-Independent Manner

**Figure 2:**
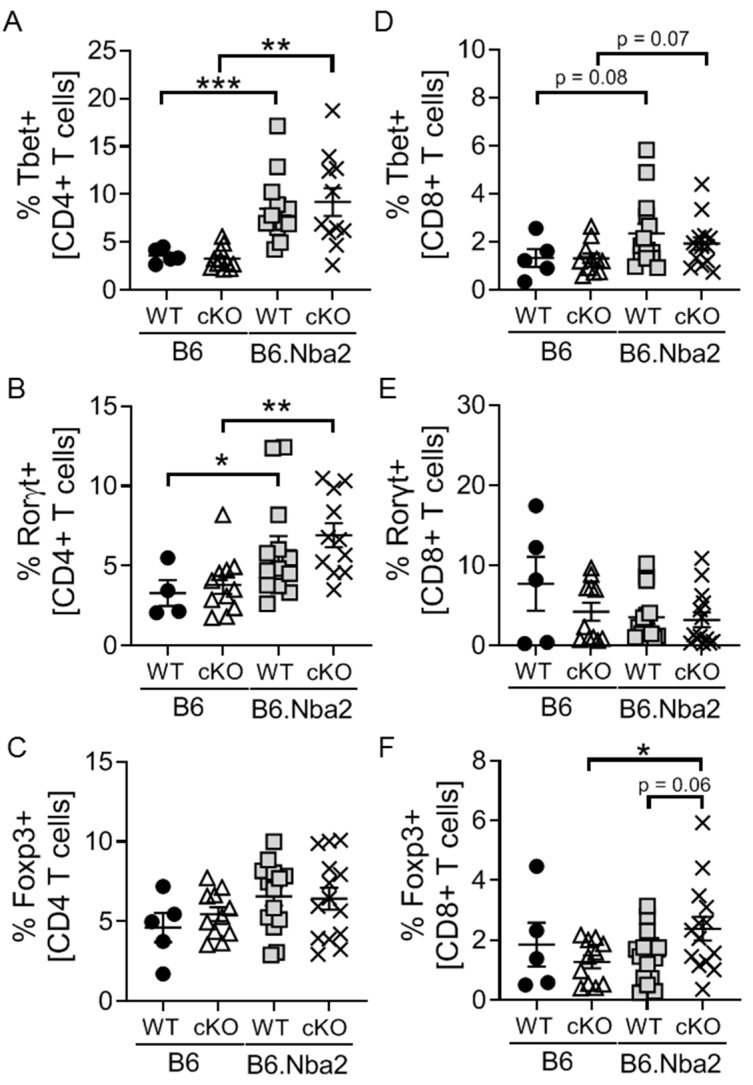
Accumulation of Th1, Th17 and Tc1 cells in B6.Nba2 mice is IFNAR independent. IFNAR-sufficient and -deficient female B6 and B6.Nba2 mice were analyzed for frequencies of CD4+ Th1, Th17 and Treg (**A**–**C**) and CD8+ Tc1, Tc17, Tcreg (**D**–**F**). Each symbol represent one mouse. *n* = 4–5 (B6), *n* = 11 (B6 cKO), *n* = 14 (B6.Nba2), *n* = 9 (B6.Nba2 cKO). * *p* < 0.05; ** *p* < 0.01; *** *p* < 0.001, Student’s unpaired *t*-test with Welch’s correction.

To further understand the contribution of T cells to lupus-like disease in the B6.Nba2 mouse model, frequencies of T helper CD4+ (Th) and T cytotoxic CD8+ [33] subsets were quantified based on the expression of master transcription factors. B6.Nba2 mice displayed significantly elevated levels of both Th1 and Th17 cells, but no significant difference in Tregs (Figure 2A–C). A trend towards elevated levels of Tc1 cells was also observed, but no changes in Tc17 cells was noted (Figure 2D,E). Interestingly, we found elevated levels of Foxp3+ Tc regulatory cells only in B6.Nba2 cKO mice (Figure 2F). 

**Figure 3:**
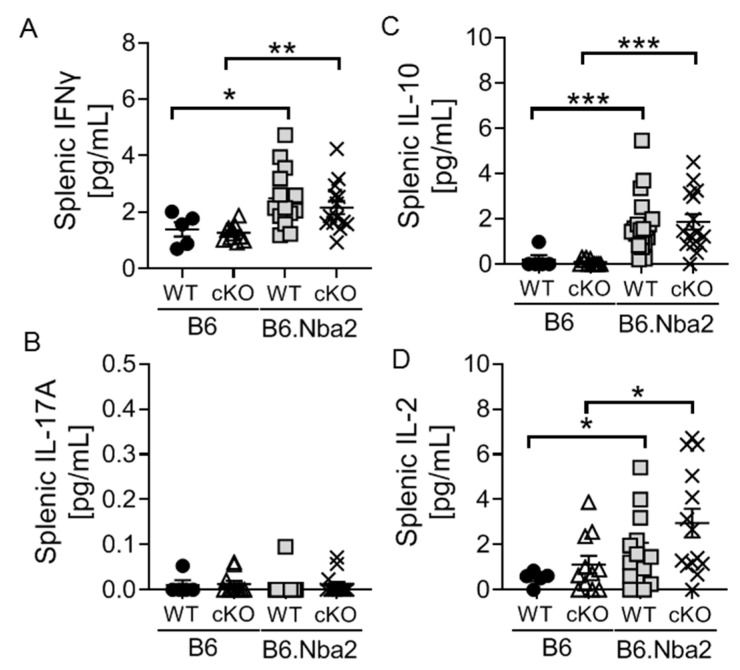
Intrasplenic levels of cytokines are dysregulated in B6.Nba2 mice independent of T-cell specific IFNAR expression. IFNAR-sufficient and -deficient female B6 and B6.Nba2 mice were analyzed for levels of intrasplenic cytokines: IFNγ (**A**), IL-17A (**B**), IL-10 (**C**), and IL-2 (**D**) as described in Materials and Methods. Each symbol represent one mouse. *n* = 4–5 (B6), *n* = 11 (B6 cKO), *n* = 14 (B6.Nba2), *n* = 9 (B6.Nba2 cKO). * *p* < 0.05; ** *p* < 0.01; *** *p* < 0.001, Student’s unpaired *t*-test with Welch’s correction.

Th1 and Th17 cells produce IFNγ and IL-17, respectively, while Tregs are particularly known for their production of IL-10 and TGFβ. Intra-splenic levels of IFNγ, IL-17 and IL-10 were measured to further understand the potential impact of dysregulation within these cell subsets. As expected IFNγ levels were significantly elevated in B6.Nba2 mice regardless of T-cell specific IFNAR expression (Figure 3A). Surprisingly, we found no significant levels of IL-17A in any of the mice (Figure 3B) despite the significantly elevated levels of Th17 cells. Intra-splenic IL-10 levels were also highly upregulated in both WT and cKO B6.Nba2 mice, as were IL-2 levels (Figure 3C,D), supporting the accumulation of regulatory T cell subsets.

### 2.3. Nba2-Driven Dysregulation of T Follicular Helper (Tfh) Cells, Germinal Center Associated B Cells and Associated Cytokines Is Independent IFNAR Expression

**Figure 4:**
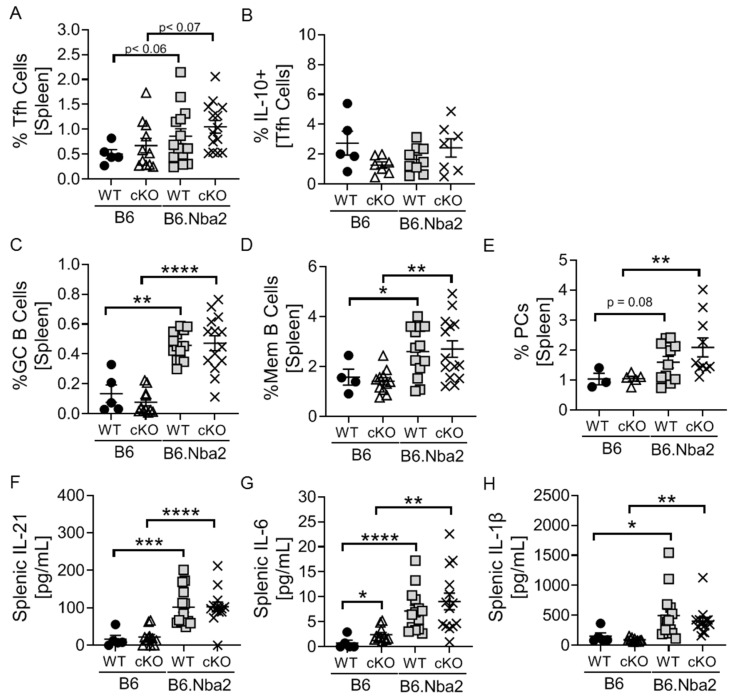
Spontaneous accumulation of Tfh cells, GC B cells, memory B cells and plasma cells is independent of T-cell specific IFNAR expression. IFNAR-sufficient and -deficient female B6 and B6.Nba2 mice were analyzed for splenic frequencies of Tfh cells (**A**,**B**), GC B cells (**C**) memory B cells (**D**), Plasma cells (**E**) by flow cytometry. (**F**–**H**) Splenic IL-21, IL-6 and IL-1β levels were determined by electrochemiluminescence as described in Material and Methods. Each symbol represent one mouse. *n* = 4–5 (B6), *n* = 11 (B6 cKO), *n* = 14 (B6.Nba2), *n* = 9 (B6.Nba2 cKO). * *p* < 0.05; ** *p* < 0.01; *** *p* < 0.001; **** *p* < 0.0001, Student’s unpaired *t*-test with Welch’s correction.

We have previously reported that T follicular helper cells (Tfh), germinal center (GC) B cells, memory B cells and plasma cells are present at elevated levels in B6.Nba2 mice [12,13]. To determine if Tfh cell accumulation was directly dependent on IFNAR expression by T cells, we tested levels in B6 and B6.Nba2 cKO mice. There were no differences in total Tfh cells or IL-10+ Tfh cells between B6.Nba2 WT and cKO mice (Figure 4A,B). Similarly, T-cell specific IFNAR deficiency did not affect levels of GC B cells, memory B cells and PCs (Figure 4C–E). Tfh cells produce IL-21, IL-6 and IL-1β, all cytokines involved in driving the germinal center reaction. As expected all three cytokines were significantly elevated in both WT and cKO B6.Nba2 mice (Figure 4F–H). We did observe a slight increase in intra-splenic IL-6 levels in B6 cKO mice, as compared to B6 WT mice; however, the significance of this observation remains unknown.

### 2.4. Nba2-Driven Splenomegaly and ANA Production Is Intact in B6.Nba2.cKO Mice

We found no IFNAR dependent difference in splenomegaly, quantified by splenocyte count and spleen weight, leaving *Nba2*-driven splenomegaly intact in the B6.Nba2 cKO mice (Figure S2A,B). Similarly, *Nba2*-driven ANA production was intact, and found to be independent of T cell-specific IFNAR expression (Figure S2C,D).

### 2.5. DP Thymocytes Accumulate in B6.Nba2 Mice in a Partially IFNAR-Dependent Manner

**Figure 5:**
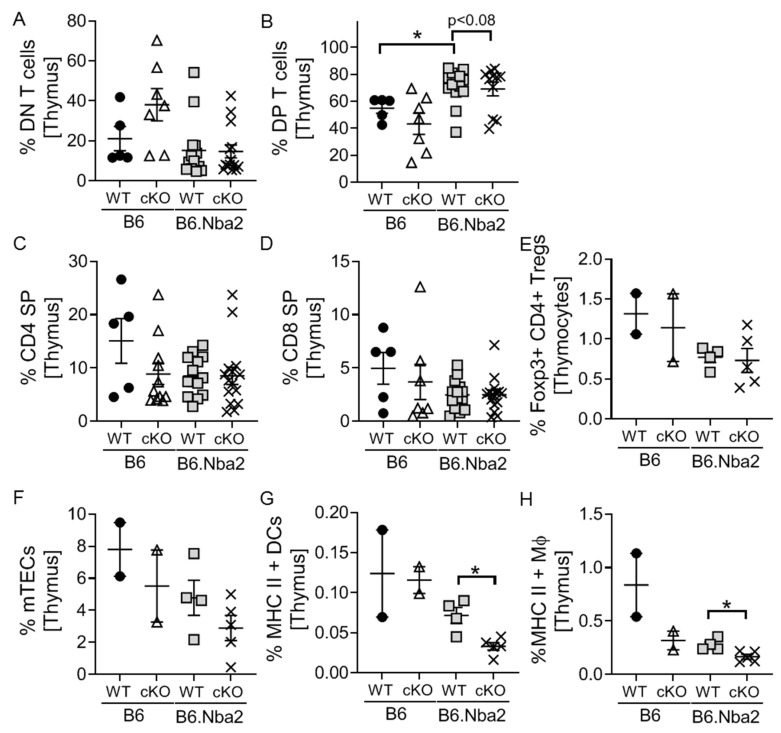
T-cell specific IFNAR deficiency partly reverses DP thymocyte accumulation and lowers levels of MHC-II-expressing thymic dendritic cells. IFNAR-sufficient and -deficient female B6 and B6.Nba2 mice were analyzed for splenic frequencies of DN (**A**), DP (**B**) and SP (**C**,**D**) thymocytes. *n* = 4–5 (B6), *n* = 11 (B6 cKO), *n* = 14 (B6.Nba2), *n* = 9 (B6.Nba2 cKO). A separate cohort of mice were further analyzed for thymic CD4+ Tregs (**E**), medullary thymic epithelial cells (mTECs) (**F**), MHC class II+ thymic DCs (**G**) and MHC class II+ thymic macrophages (**H**). Each symbol represent one mouse. * *p* < 0.05, Student’s unpaired *t*-test with Welch’s correction.

In order to determine if thymocyte development was altered by the inability to signal through IFNAR, we quantified total thymocytes and the four major subsets of developing thymocytes. No significant differences were found in total thymocytes (data not shown). Similarly, there was no change in the frequency of DN thymocytes (Figure 5A). DP thymocytes were significantly elevated in WT B6.Nba2 mice and the numbers were partly reversed in B6.Nba2 cKO mice, although the changes did not reach statistical significance (*p* = 0.06) (Figure 5B). Interestingly, neither CD4+ nor CD8+ SP thymocytes were significantly altered in WT B6.Nba2 mice, although both trended lower than WT B6 mice (Figure 5C,D). A smaller cohort of mice were further analyzed for levels of thymic Foxp3+CD4+ Tregs, and while levels trended lower in B6.Nba2 mice, no difference was found between WT and cKO B6.Nba2 mice (Figure 5E). Finally, as the development of T cells depend on proper levels of MHC expressing subsets including medullary thymic epithelial cells (mTECs), dendritic cells (DCs) and macrophages (Mϕ), we tested these and found to our surprise significantly reduced levels of MHC-II expressing DCs and Mϕs in B6.Nba2.cKO mice as compared with WT B6.Nba2 mice (Figure 5F–H).

### 2.6. Kidney-Infiltrating T Cells Are Unaffected by Intrinsic IFNAR Expression

**Figure 6:**
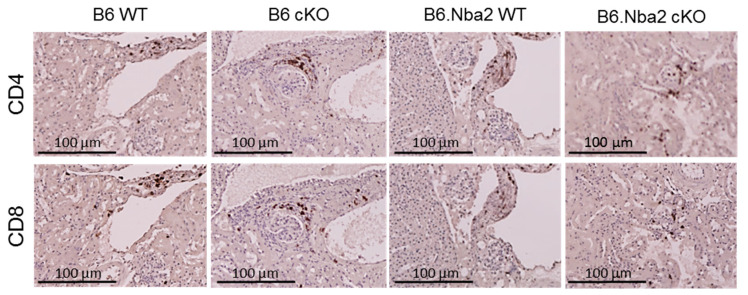
CD4+ and CD8+ T cells infiltrate kidneys independent of T-cell specific IFNAR expression. Kidneys were stained for CD4 (top row) and CD8 (bottom row) and counterstained with hematoxylin (light bluish-brown). CD4 and CD8 staining appears in dark brown. Representative images are shown. B6: *n* = 2, B6 cKO: *n* = 2 B6.Nba2: *n* = 4, B6.Nba2 cKO: *n* = 5.

Finally, both CD4+ and CD8+ T cells have been shown to infiltrate kidneys of lupus patients. In particular, CD8+ T cells are believed to contribute to nephritis in SLE patients as levels associate strongly with both proteinuria and serum anti-dsDNA autoantibodies [45]. We stained kidneys from WT and cKO B6 and B6.Nba2 mice for CD4 and CD8 to visualize levels of infiltrating T cell populations (Figure 6). Both CD4+ and CD8+ T cells were strongly represented among interstitium infiltrating cells of all strains, while sporadic periglomerular infiltration was observed predominantly in cKO mice of both strains.

## 3. Discussion

Systemic lupus erythematosus (SLE) is an autoimmune disorder characterized by dysregulated T and B cells. The disease has been associated with increased IFN-I stimulation of immune cells and gene expression analyses have shown strong interferon-stimulated gene signatures within PBMCs [31,32]. Most animal models have similarly shown dependency on functional type I interferon receptor (IFNAR) expression. As such, we have previously described that global IFNAR-deficient B6.Nba2 mice are protected from disease development [25]. Furthermore, the B6.Nba2 mouse model of lupus develops a disease dependent on CD4+ T cells, as MHC-II-deficient mice are protected from disease [16], suggesting that IFN-I stimulation of CD4+ T cells could be involved in disease pathogenesis. Furthermore, IFN-I stimulation is known to promote T cell survival and induce the expression of Tfh markers such as *Bcl6*, *Cxcr5* and *Pdcd1* [33,37].

We here report that WT B6.Nba2 mice display a strong CD4+ T cell phenotype with significantly elevated levels of activated and differentiated CD4+ T cells, a strong presence of Th1, Th17 and Tfh cells, and significantly elevated intra-splenic levels of T cell associated cytokines, such as IFNγ, IL-2 and IL-21. Surprisingly, however, neither of these phenotypes were found to depend on T cell-specific IFNAR expression. While currently unknown, it is plausible that a lack of effect in cKO mice is due to the presence of compensatory mechanisms by IFNγ or other T cell effector cytokines activated in these mice. It should also be noted that while IFNα has been suggested to be the main IFN-I driving lupus-like disease, other members of the IFN-I family, such as IFNβ, also signals via IFNAR. This is notable because in a model of multiple sclerosis, IFNβ was found to facilitate apoptosis of Th17 cells [46], thus potentially countering IFNα-driven cell survival.

Among the few differences observed between B6.Nba2 WT and cKO mice was a trend towards elevated levels of Foxp3+CD8+ Tc regulatory cells and a reduction in CD44^high^CD62L^low^ effector CD8+ T cells in B6.Nba2 cKO mice. The presence of elevated levels of CD44^high^CD62L^low^ CD8+ T cells in B6.Nba2 mice is consistent with a role for IFN-I in promoting CD8+ T cell activation, potentially via cross-priming as previously reported [36]. Suppressive CD8+ T cells have previously been found to functionally inhibit effector T cell function via secretion of IL-10 [47]. The cells are either CD28+ or CD28-, but typically co-express Foxp3+, CD62L+ and/or PD-1+ [41]. In animal models, suppressive CD8+ T cells were first identified in NZW/BXSB mice as treatment with depleting anti-CD8 antibodies resulted in exacerbated renal disease and decreased survival [48]. Reduced levels of suppressive CD8+ T cells were also observed in (NZB×NZW)F1 mice, while elevated levels were found in lupus-prone mice tolerized in response to peptide treatment [41]. 

We observed increased periglomerular infiltration by CD4+ and CD8+ T cells in a small cohort of cKO mice; however, whether these cells represent effector or regulatory T cell subsets remain unknown. Suppressive CD8+ T cells have been found to produce IL-10, IFNγ and TGFβ, all of which are required for their suppressive function [49], potentially via autocrine regulation of Foxp3 [40]. We observed elevated intra-splenic levels of both IL-10 and IFNγ in B6.Nba2.cKO mice; however, neither cytokine was present at levels significantly different between WT and cKO B6.Nba2 mice. It is possible that a difference in cytokine production by Foxp3+CD8+ cells is concealed by the high levels of these cytokines produced by CD4+ T cell subsets and thus further analyses determining intracellular levels in each cell subset are needed to firmly establish their cytokine profile. We did not determine levels of TGFβ levels in the mice and did not examine levels of cytokines in kidney eluates.

B6.Nba2 mice present with an accumulation of DP thymocytes and reduced levels of both CD4+ and CD8+ SP cells. It remains unknown how IFN-I stimulation of DP thymocytes affect T cell maturation; however, it is conceivable that thymocyte survival may be prolonged as previously seen for mature T cells in vitro [33]. Alternatively, processes involving dysregulated thymic MHC-expressing cell subsets such as mTECs, DCs and Mϕs may be involved via a feedforward loop, as we observed reduced levels of these cells in B6.Nba2 cKO mice. Further studies evaluating the cytokine release response of DP thymocytes to IFN-I stimulation and the subsequent effect(s) on surrounding antigen presenting cell subsets are needed to further explore this possibility.

Finally, it has been reported that the presence of exhausted CD8+ T cells associate with better clinical outcomes in SLE patients [38]. While the surface pattern of exhausted CD8+ T cells is well established for human-derived cells, a comprehensive analysis of such cells in mouse models remains to be conducted. IFN-I stimulation of CD8+ T cells have been found to promote activation, cytolytic activity and cross-priming [50]. Given the elevated levels of CD44^high^CD62L^low^ effector CD8+ T cells observed in B6.Nba2 WT mice, but not in B6.Nba2 cKO mice, it is possible that these cells represent IFN-I stimulated non-exhausted pathogenic cells. Further studies are needed to determine the functional role of these cells in B6.Nba2 mice.

In conclusion, T cell-specific IFNAR-deficiency in the B6.Nba2 mice did not significantly affect lupus-like disease development. Surprisingly, lupus-associated dysregulation of the CD4+ T cell compartment was independent of direct IFN-I stimulation. Small alterations in the levels of CD44^high^CD62L^low^ effector CD8+ T cells and Foxp3+ CD8+ regulatory Tc cells were observed in B6.Nba2 cKO mice as compared with WT littermates; however, these changes were not enough to significantly alter disease pathogenesis in the model.

## 4. Materials and Methods

### 4.1. Animals

B6.Nba2 mice (B6.Nba2.ABC line [43] were bred in-house. B6(Cg)-Ifnar1tm1.1Ees/J (The Jackson Laboratories, strain #028256), C57BL/6 (B6), and B6.Cg-Tg(Cd4-cre)ICwi/Bflu.J; (The Jackson Laboratories, strain #022071) were purchased from JAX-mice. T-cell specific IFNAR-deficient mice (cKO) were generated on B6 and B6.Nba2 backgrounds via backcrossing as previously described [36,51] using the following primers: IFNAR5′: 5′ TGC TTT GAG GAG CGT CTG GA 3′ IFNAR3′: 5′ CAT GCA CTA CCA CAC CAG GCT TC 3′ IFNARΔ5′: 5′ TAG CCC CAG GGT AGT TAA CTC TTG A 3′ CD4 F: 5′ GTT CTT TGT ATA TAT TGA ATG TTA GCC 3′ CD4 R: 5′ TAT GCT CTA AGG ACA AGA ATT GAC A 3′ CD4ΔR: 5′ CTT TGC AGA GGG CTA ACA GC 3′. Immediately prior to euthanasia at 4 months of age, all study mice were bled for isolation of serum. This study used only female mice. All experimental mice were housed in specific pathogen-free housing at Lerner Research Institute and all studies were IACUC approved.

### 4.2. Flow Cytometry

Freshly isolated splenocytes and thymocytes were stained for flow cytometry. Intracellular staining was performed using the FOXP3 intracellular staining kit reagents (ThermoFisher, Waltham, MA, USA) and all intracellular antibodies were run alongside an appropriate isotype control. The following antibodies were used for staining and, unless otherwise noted, sourced from eBioscience (San Diego, CA, USA). anti-B220 IgG (Clone RA3-6B2), anti-Bcl6 IgG (Clone BCL-DWN), anti-CD3 IgG (Clone 145-2C11), anti-CD4 (Clone GK1.5), anti-CD8 IgG (Clone 53-6.7), anti-CD11b IgG (Clone M1/70), anti-CD11c (Clone N418), anti-CD16/32 (Clone 93), anti-CD19 (Clone 1D3), anti-CD21/35 IgG (Clone 4E3), anti-CD23 IgG (Clone B3B4), anti-CD25 IgG (Clone PCL61.5), anti-CD38 IgG (Clone 90), anti-CD40 IgG (Clone HM40-3), anti-CD40L IgG (Clone MR1) anti-CD43 IgG (Clone R2/60), anti-CD44 IgG (Clone IM7), anti-CD62L IgG (Clone MEL-14), anti-CD69 IgG (Clone H1.2F3), anti-CD93 IgG (Clone AA4.1), anti-CD138 IgG (Clone 281-2), anti-CXCR5 IgG (Cat:551960 (BD Pharmigen, San Diego, CA, USA), anti-F4/80 IgG (Clone BM8), anti-FoxP3 IgG (Clone FJK-16s), anti-GL7 IgG (Clone GL-7), anti-Gr1 IgG (Clone RB6-8C5), anti-IgD IgG (Clone 11-26c), anti-IgG1κ IgG (Clone eBRG1), anti-IgG2aκ IgG (Clone eBR2a), anti-IgG2bκ IgG (Clone eB149/10H5), anti-IgM IgG (Clone 11/41), anti-IL-10 IgG (Clone JES5-16E3), anti-IL-4 IgG (Clone 11B11), anti-Ly6C IgG (Clone AL-21), anti-Ly6G IgG (Clone 1A8), anti-MHC-II IgG (Clone M5114.15.2), anti-PDCA IgG (Clone 927), anti-PD-1 IgG (Clone J43), anti-Rorγt IgG (Clone AFKJS-9), anti-Siglec H IgG (Clone 440c), anti-SignR1 IgG (Clone 22D1), anti-Tbet IgG (Clone eBio4B10). Streptavidin Conjugated Antibodies were used with catalog numbers: 45-4317-82, 12-4317-87, and 405208 (Biolegend, San Diego, CA, USA). Flow cytometry was run on a BD LSR Fortessa™ flow cytometer (BD Biosciences, San Jose, CA, USA) and BD FACSDiva™ software (BD Biosciences, San Jose, CA, USA). FlowJo Version 10 Software (FlowJo, Ashland, OR, USA) was used for analysis. Gating strategies for all cell populations are provided in Figure S3 or in Keller et al., 2021 [13].

### 4.3. Intra-Splenic Cytokine Levels Using Electrochemiluminescence

Cytokine concentrations in the spleen supernatant of the cKO mice and corresponding controls were quantified using a custom U-PLEX mouse biomarker assay from Mesoscale Discovery (Meso Scale Discovery LLC, Rockville, MD, USA). Spleen supernatant was harvested from freshly isolated splenocytes by incubating total single cell splenocytes at 37 °C for 10 min in 1×PBS to allow for cytokine secretion, and the supernatant containing cytokines was isolated using centrifugation. Spleen supernatants were immediately stored at −80 °C until the assay was performed. The assay was run according to the manufacturer’s directions. Briefly, antibody and linkers were coupled, the plate was coated and undiluted spleen supernatant and calibrators were applied. Sulfo-tagged antibodies were applied according to manufacturer’s instructions and the concentration was measured by light emission using the Meso QuickPlex 120 SQ (Meso Scale Discovery LLC, Rockville, MD, USA).

### 4.4. Anti-dsDNA Autoantibody ELISA

Serum concentrations of anti-dsDNA IgG were determined using ELISA kits (Alpha Diagnostics International, San Antonio, TX, USA) and run according to the manufacturer’s protocol. Samples were diluted 1:100. IgG subtype ELISAs were developed with anti-IgG subtype specific-HRP antibodies (IgG1, IgG2b, IgG2c, IgG3) (all from Southern Biotech) as previously published [13].

### 4.5. Immunohistochemistry

Paraffin-embedded kidney sections (5 µm) were chemically deparaffinized by incubating 2× in Clear Rite 3′ (ThermoFisher, Waltham, MA, USA). Tissue was re-hydrated by washing twice each in Flex 100 and Flex 95 (ThermoFisher, Waltham, MA, USA), and tap water. Tissue was isolated on slides using a hydrophobic pen and ~100 μL HBSS 2% FBS was applied to block tissue for 30 min, then removed. Anti-CD4 or Anti-CD8 antibodies (Abcam) were diluted 1:1500 in blocking solution and added to the tissue for incubation overnight at RT in a humidity chamber. The next day slides were washed with 1×PBS and horse-radish peroxidase (HRP)-conjugated rabbit anti-rodent antibody (RMR622H, Biocare Medical, Pacheco, CA, USA) was applied for 20 min. Slides were washed in phosphate buffered saline (PBS) and DAB (3,3′-diaminobenzidine) substrate (Biocare Medical, Pacheco, CA, USA) was applied for one minute and then washed in water. Slides were counterstained with hematoxylin 7211 (ThermoFisher, Waltham, MA, USA) for one minute, followed by clarifier, and a bluing reagent (ThermoFisher, Waltham, MA, USA). Finally, slides were washed in three rounds of ethanol at increasing concentrations (70%, 90%, and 95%) and finished with incubation in Clear Rite (ThermoFisher, Waltham, MA, USA) before mounting media was applied to finish the staining.

### 4.6. Microscopy

All imaging was conducted on an “All in One” BZ-X series Keyence microscope and analyzed using the BZ-X Keyence Analysis software (Keyence, Osaka, Japan).

### 4.7. Statistic

Two-way comparisons between groups of mice were conducted using Student’s unpaired t-test with Welch’s correction.

## Data Availability

Data is contained within the article or Supplementary Material.

## Supplementary Materials

The following are available online at https://www.mdpi.com/article/10.3390/ijms23020874/s1.
