# Supplementary material for: Spontaneous CD4+ T Cell Activation and Differentiation in Lupus-Prone B6.Nba2 Mice Is IFNAR-Independent
Source: Int J Mol Sci. 2022 Jan 14;23(2):874. doi: 10.3390/ijms23020874 (PMC8778657; doi:10.3390/ijms23020874)
Supplement: Supplementary file 1 [file ijms-23-00874-s001.zip › ijms-1514120-supplementary.pdf]

International Journal of *Molecular Sciences*

MDPI

Supplementary Materials

Spontaneous CD4+ T Cell Activation and Differentiation in Lupus-Prone B6.Nba2 Mice is IFNAR-Independent

Emma J. Keller [1,2], Nina Dvorina [2] and Trine N. Jørgensen [2,*]

[1] Department of Molecular Medicine, Cleveland Clinic Lerner at Case Western Reserve University, 44195 Cleveland, OH, USA; ejk114@case.edu

[2] Department of Inflammation and Immunity, Lerner Research Institute, Cleveland Clinic, 44195 Cleveland, OH, USA; dvorinn@ccf.org

* Correspondence: jorgent@ccf.org; Tel.: +1-216-4447454

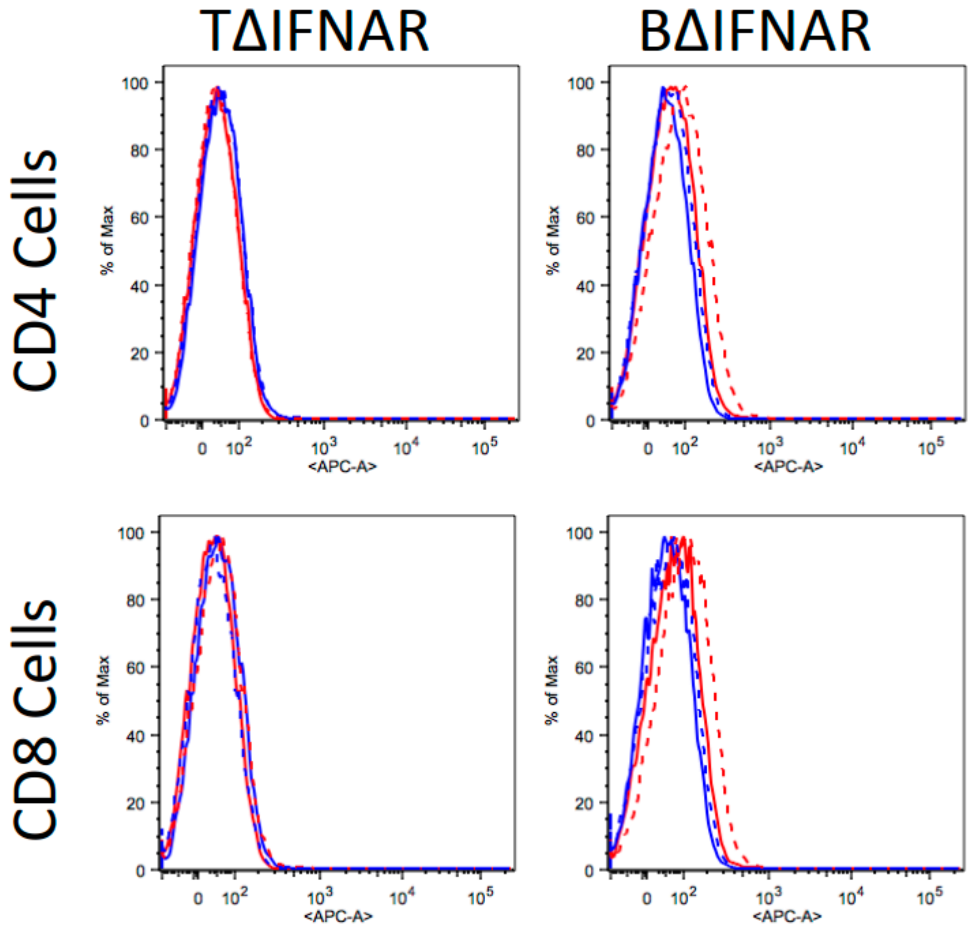

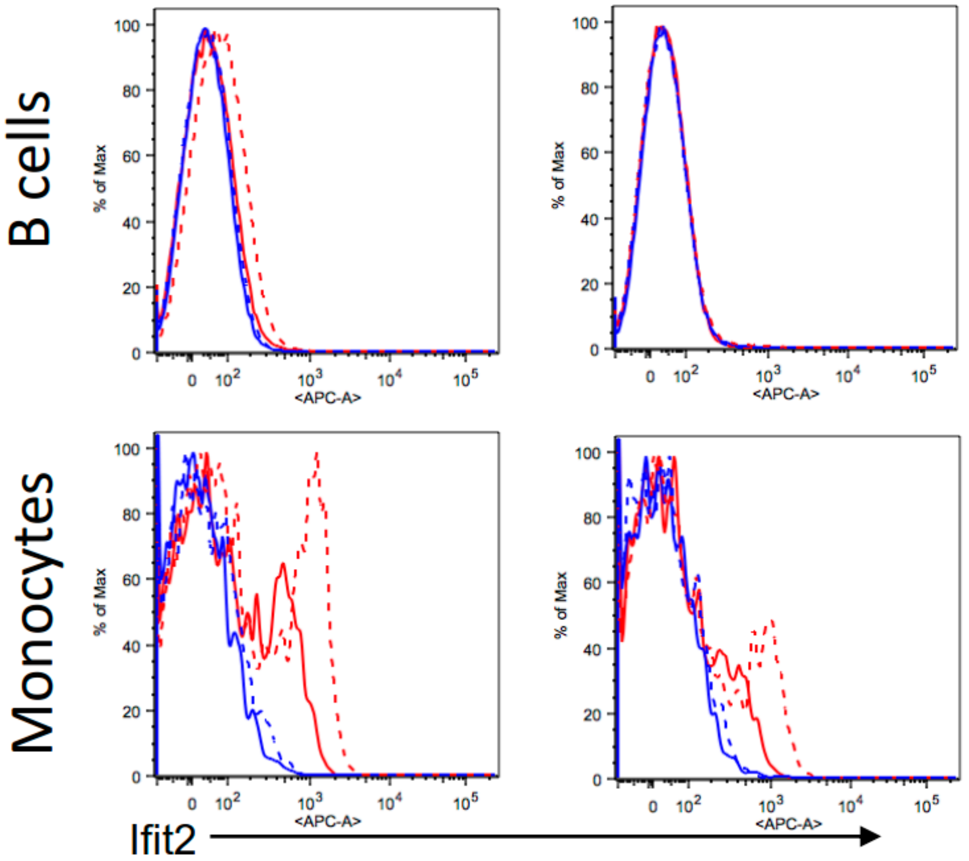

(**A**) (**B**)

Figure S1. Ifit2 is not induced in response to recombinant IFNalpha in CD4+ and CD8+ T cells from B6.Nba2.TΔIFNAR mice. Splenocytes were isolated from B6.Nba2.TΔIFNAR (column **A**) and B6.Nba2.BΔIFNAR (column **B**)[13], and stimulated ex vivo with recombinant mouse IFNαA (stippled lines) or control 1×PBS (solid line) for 16 hours. Splenocytes were surface stained for CD4, CD8, CD11b and B220, and intracellular levels of Ifit2 were determined via staining with anti-Ifit2 antibody in the presence (red line) or absence (blue line) or secondary fluorescently labeled anti-rat IgG.

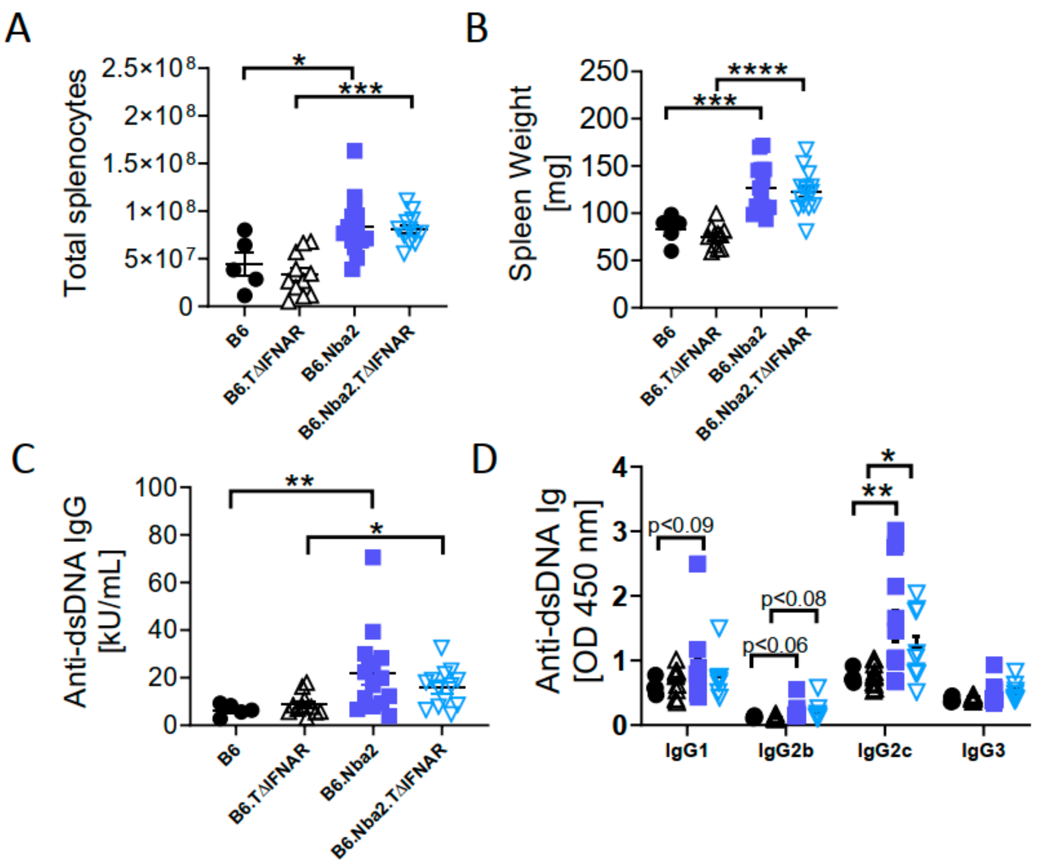

Figure S2. Splenomegaly and autoantibody production is intact in B6.Nba2.TΔIFNAR mice**.** Splenomegaly was determined by measuring splenocyte count (**A**) and spleen weight (**B**) at 4 months of age. Presence of anti-dsDNA IgG and anti-dsDNA IgG subtypes in serum were measured by ELISA (**C**,**D**). B6: $n = 5$, B6. TΔIFNAR: $n = 11$ B6.Nba2: $n = 13$, B6.Nba2.TΔIFNAR: $n = 14$,. Each symbol represents one mouse and data are shown as Mean ± SEM. * $p < 0.05$; ** $p < 0.01$; *** $p < 0.001$, **** $p < 0.0001$; Student's unpaired t-test with Welch's correction.

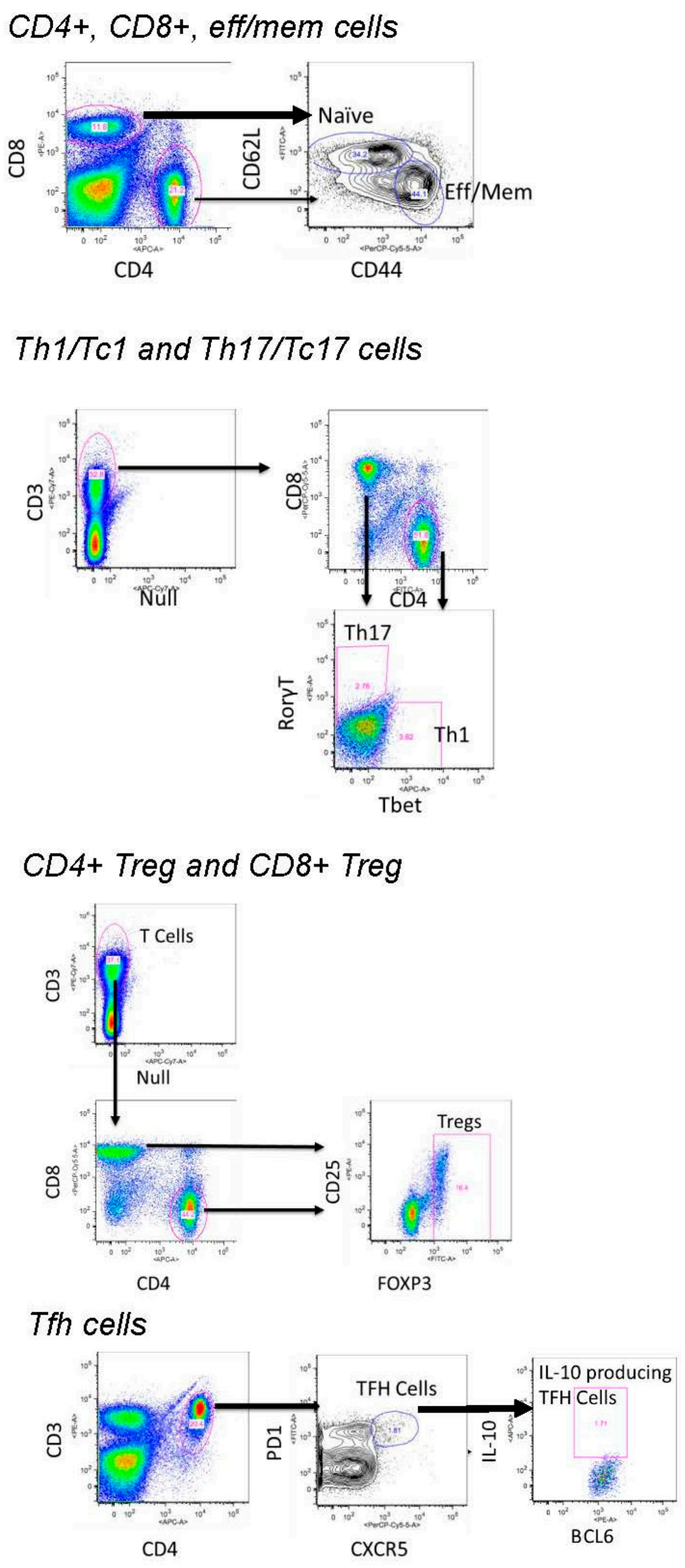

Figure S3. Gating strategies for T cell and thymic cell populations. All samples were gated for live cells using forward and side scatter properties. Gating strategies for splenic B cell subsets can be found in Keller et al [13].
